# The Effect of Local Anesthetics on Neutrophils in the Context of Different Isolation Techniques

**DOI:** 10.3390/biomedicines11082170

**Published:** 2023-08-02

**Authors:** Sara Sixt, Michael Gruber, Gesche Kolle, Thies Galla, Diane Bitzinger

**Affiliations:** Department of Anesthesiology, University Hospital Regensburg, 93042 Regensburg, Germany

**Keywords:** neutrophils, PMNs, isolation, local anesthetics, PMN functions, lidocaine, bupivacaine, levobupivacaine, ropivacaine

## Abstract

Various functions of polymorphonuclear neutrophils (PMNs) are related to diseases and postoperative plasma changes. The influence of some local anesthetics (LAs) on PMNs obtained by conventional isolation methods and their functions has already been demonstrated. This study investigates the effect of selected LAs on PMNs, comparing a new isolation method with conventional ones. To obtain the PMNs, we performed either gelafundin sedimentation, hypotonic lysis or density gradient centrifugation. Subsequently, PMNs were mixed with different concentrations of bupivacaine, levobupivacaine, lidocaine or ropivacaine. Live cell imaging and flow cytometry were performed to quantify the migration, ROS production, NETosis and antigen expression of PMNs. We found the inhibition of chemotaxis and ROS production by LAs. PMNs showed a strong reduction in time to half maximal NETosis in response to bupivacaine and lidocaine, but not to levobupivacaine and ropivacaine. We also found distinct differences in survival time and migration duration between the isolation methods. This suggests that the careful selection of LAs has a short-term impact on in vitro PMNs.

## 1. Introduction

### 1.1. Polymorphonuclear Neutrophils: Immune Mechanisms in Disease

In humans, neutrophils, also known as polymorphonuclear cells (PMNs), make up the largest portion of leukocytes and are therefore an essential component of the blood [1]. As part of the innate immune system, their primary job is to fight off pathogens in the body. In order to fulfill this task, targeted migration (chemotaxis), reactive oxygen species (ROS) production, phagocytosis and formation of neutrophil extracellular traps (NETs) are defense mechanisms used by PMNs [1,2,3,4].

In NETosis, a self-destruction program, PMNs release NETs consisting of DNA and proteins such as histones, myeloperoxidase and neutrophil elastase [5,6]. These net-like NETs can immobilize pathogens and render them harmless with the help of the adhering proteins. Several studies have shown that there is an association between misguided NETosis and various diseases. For example, elevated levels of citrullinated histone H3, which is a specific marker for the presence of NETs, have been detected in COVID-19 infection [7] and various tumor identities [8,9,10]. In addition, NETs are associated with other tumor types and metastases [11,12,13,14].

It is known that surgeries also elevate the plasma level of NETosis markers [15]. For instance, plasma from patients after cardiopulmonary bypass surgery shows higher NET levels [16].

### 1.2. Local Anesthetics: Their Usage and Influence on the Immune System

Local anesthetics (LAs) are commonly used analgesics for everyday clinical practice, especially in perioperative settings and cancer [17,18]. 

They exert their main effect on neurons by inhibiting the influx of sodium ions [17,18]. In addition, they also affect granulocytes, which is why they have been considered as therapeutic agents in inflammatory situations [19,20,21]. However, it has not yet been sufficiently clarified how LAs affect PMNs. So far, lidocaine and bupivacaine, besides some other LAs, have been shown to inhibit ROS production [22,23,24] as well as the adhesion, phagocytosis and migration [22,24,25] of granulocytes. Moreover, the point of maximal ROS production and half-maximal NETosis was reached more rapidly with lidocaine or bupivacaine [25]. Many studies emphasize the importance of dosage with regard to impact on PMN functions [22,24,25]. We evaluated the effects of LA concentrations ranging from 0 to 13 mmol/L. Conflicting results are published for surface antigen expression. In one study, lidocaine and bupivacaine decreased the expression of CD11b, which is important for adherence and Diapedesis as part of the Mac-1 [24]. In another study, both LAs had no effect on the expression of CD11b, CD62L and CD66b [25]. CD62L as a L-selectin is important for rolling [26,27]. In contrast, ropivacaine showed no effects on phagocytosis, oxidative burst or CD11b expression [24].

In addition, racemic bupivacaine, R- and S-bupivacaine have been shown to differ in their effect on surface receptor expression, phagocytosis and ROS production, as well as on neutrophil priming [28,29]. S-bupivacaine shows a lesser suppressing effect on Fcγ-receptor and CD35 expression, phagocytosis and ROS production than racemic bupivacaine and R-bupivacaine [29].

### 1.3. Study Hypothesis: Impact of LA on PMNs 

The studies mentioned above show that LAs influence various functions of granulocytes. The aim of this study is to investigate the impacts of lidocaine, bupivacaine, levobupivacaine and ropivacaine on the migration, ROS production, NETosis and antigen expression of PMNs isolated with three different techniques. For this purpose, we used live cell imaging and flow cytometry. Special attention was paid to the effects of lidocaine, bupivacaine and levobupivacaine on most native PMNs isolated with gelafundin sedimentation. In line with the result of Welters et al. [29], we hypothesize that racemic bupivacaine has a higher impact on PMNs than levobupivacaine. PMNs were isolated with different methods to check the impact of centrifugation steps on the PMNs. 

## 2. Materials and Methods

### 2.1. Experimental Design and Observed Parameters

**Figure 1:**
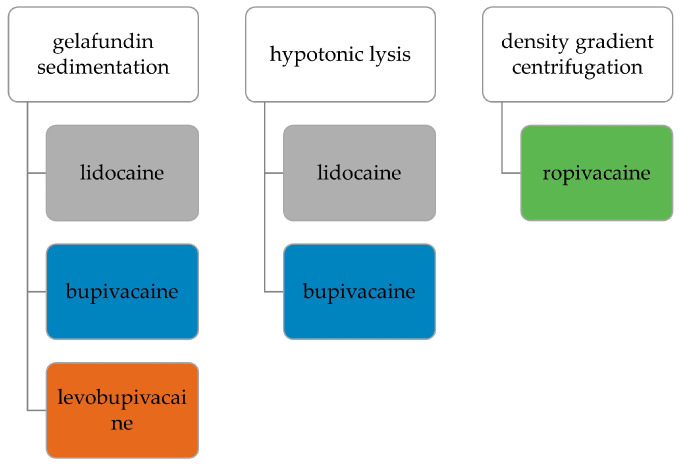
Tested combinations of PMN isolation techniques and LAs used for incubation.

This study was arranged in two steps: First, the concentration and time-dependent effect of LAs on PMNs were investigated. Therefore, we used the LAs lidocaine, bupivacaine and levobupivacaine on PMNs obtained via gelafundin sedimentation, lidocaine and bupivacaine on PMNs isolated via hypotonic lysis and ropivacaine on PMNs isolated via density gradient centrifugation, as displayed in Figure 1. This setup was chosen to investigate whether the effects of LAs and centrifugation detected in the different isolation methods are additive or independent [25,30].

We observed the parameters presented in Table 1. For migration analysis, we evaluated the absolute and relative (r) values of track length (TL), track displacement along the chemotactic gradient (TDX) and track duration (TD). For this purpose, 30 min sections were analyzed. The 30 min sections were sorted into three time periods (first: 0–10 h, second: 10–15 h; third: 15–21 h). Tracks with TD > 900 s and TL > 25 µm were included for evaluation. For NETosis, two parameters were calculated: first, the times for half-maximal NETosis (ET_50_NETosis) at specific LA concentrations and second, the concentration to reach half-maximal reduction in ET_50_NETosis for each LA (EC_50_NETosis).

For PMNs obtained via hypotonic lysis or density gradient centrifugation, we only observed migration and NETosis parameters.

In a second step, we compared the results of the three isolation techniques regarding the parameters for migration and NETosis of PMNs treated with or without LAs.

### 2.2. Blood Sampling

With the written consent of 77 healthy *human* volunteers, 7.5 mL of whole blood was withdrawn in serum clot activator and lithium heparin-anticoagulated collection tubes. This was approved by the local ethics committee of the University of Regensburg (vote: 12–101-0192, date of approval: 16 August 2012).

### 2.3. Collection of Autologous Serum and Isolation of PMNs

Autologous serum was obtained via centrifugation of the serum tube for 10 min at 1181× *g* and room temperature. 

For isolation of the PMNs, gelafundin sedimentation, hypotonic lysis or density gradient centrifugation were performed as described in the following.

#### 2.3.1. Gelafundin Sedimentation

A mixture of lithium-heparinized whole blood and 10% gelafundin (Gelafundin^®^ ISO 40 mg/mL infusion solution) (B. Braun SE, Melsungen, Germany) was prepared. After 30 min, during which sedimentation of the erythrocytes took place, the supernatant containing the PMNs was removed. A 1:2 dilution of the recovered supernatants with autologous serum was carried out for the test series with the 3D-µ-slides. No dilution was carried out for the experiments with the µ-Slides VI 0.1 and flow cytometry.

#### 2.3.2. Hypotonic Lysis

Isolation was carried out as described by Kolle et al. with distilled aqua and sodium chloride solution (Merck, Darmstadt, Germany) [25]. During this procedure, cells were centrifuged for 5 min at 425× *g*. Subsequently, the cells were diluted to a concentration of 18 million cells/mL with RPMI (PAN-Biotech GmbH, Aidenbach, Germany) and fetal calf serum (FCS, Sigma Aldrich, Steinheim, Germany).

#### 2.3.3. Density Gradient Centrifugation

Successively, 3 mL of LeukoSpin^®^ medium (pluriSelect Life Science Leipzig, Germany), 3 mL of PBMC Spin^®^ Medium (pluriSelect Life Science, Leipzig, Germany) and 3 mL of lithium-heparinized whole blood as the final layer were stacked in a 15 mL tube. After centrifugation at 756× *g* for 20 min without braking, using the BioFuge™ Stratos™ (Thermo Fisher Scientific, Waltham, MA, USA), the granulocyte-rich layer was removed according to the manufacturer’s instructions and diluted with 3 mL DPBS^®^ (Dulbecco’s phosphate-buffered saline modified without calcium, magnesium and chloride, Thermo Fisher Scientific, Agawam, MA, USA). For further usage, cells were then diluted to a concentration of 18 million cells/mL with RPMI and FCS and centrifuged for 5 min at 272× *g*.

### 2.4. Local Anesthetics and Fluorescence Staining

The LAs lidocaine (lidocaine hydrochloride monohydrate, Sigma Aldrich, Steinheim, Germany) and ropivacaine (ropivacaine hydrochloride monohydrate, Fagron, Barsbüttel, Germany) were used at final concentrations between 0–13 mM, and bupivacaine (bupivacaine hydrochloride monohydrate, Sigma Aldrich, Steinheim, Germany) and levobupivacaine (levobupivacaine hydrochloride, Sigma Aldrich, St. Louis, MO, USA) were used at concentrations between 0 and 3 mM. The concentrations were based on clinically used doses. They were categorized into 3 concentration groups (control: 0 mM; low: lidocaine: 2.1–3.5 mM, ropivacaine and bupivacaine and levobupivacaine: 0.3–1.2 mM; high: lidocaine: 8–13 mM, ropivacaine: 1.8–13 mM, bupivacaine and levobupivacaine: 1.8–3 mM). As fluorescent dyes or leuco dyes, 4′,6-diamidine-2-phenylindole (DAPI, 0.5 µg/mL, Sigma Aldrich, Steinheim, Germany), dihydrorhodamine 123 (DHR-123, 1 μM, Molecular Probes Inc., Eugene, OR, USA) and allophycocyanin (APC) bound to MPO antibodies as a ready-to-use solution (anti-MPO, Miltenyi Biotec B.V. & Co. KG, Bergisch Gladbach, Germany) were used. Dihydrorhodamine 123 (DHR) is converted to fluorescent rhodamine 123 by reactive oxygen species. The fluorescent 4′,6-diamidine-2-phenylindole (DAPI) attaches to DNA.

### 2.5. Live Cell Imaging

For live cell imaging, the cell solutions were diluted to a final concentration of approximately 3 × 10^6^ cells/mL according to the manufacturer’s specifications.

#### 2.5.1. Migration Assay after Gelafundin Sedimentation

**Figure 2:**
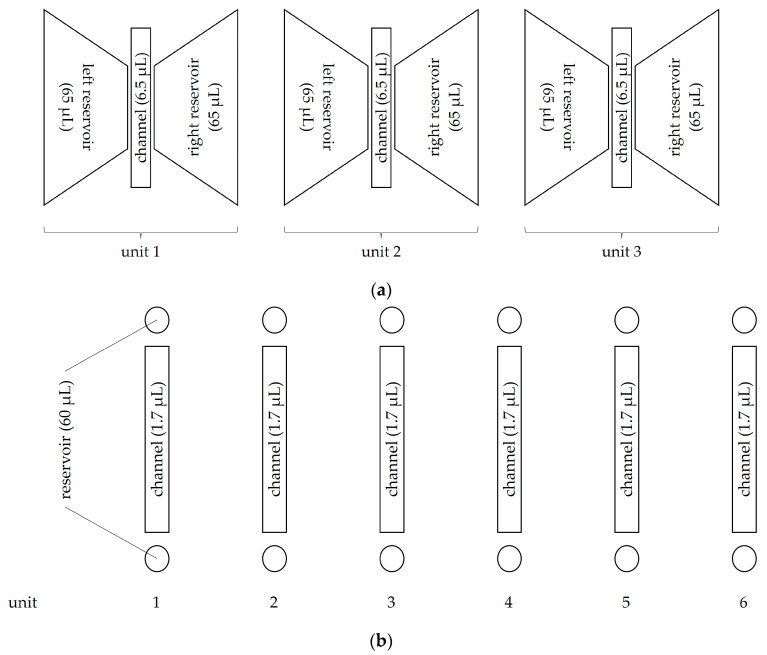
Schematic representation of (**a**) 3D-µ-slide and (**b**) µ-Slides VI 0.1. The 3D-µ-slide consists of three equal units, each with a central channel containing 6.5 µL and a reservoir to the left and right of it. Each of the reservoirs contains 65 µL. The µ-Slides VI 0.1 consists of six equal units (numbers 1–6), each with a central channel containing 1.7 µL and a reservoir above and below it. Each reservoir contains 60 µL. In both types of slides, there is no barrier between the reservoirs and the associated channel of the facility.

For migration analysis after gelafundin sedimentation, a 3D-µ-slide (Ibidi GmbH, Graefelfing, Germany) was used, consisting of three equal units, each with a central channel and a reservoir to the left and right of it (Figure 2). The 3D-µ-slide was filled as previously described in our department by Hundhammer et al. [30] with an addition of the required LA concentration in the left reservoir.

We also performed some experiments with the same isolation and procedure without LA and with FCS instead of autologous serum. If not mentioned in particular, the results of experiments with gelafundin sedimentation refer to the experiments without FCS.

#### 2.5.2. NETosis and ROS Assay after Gelafundin Sedimentation

For NETosis and ROS production analysis after gelafundin sedimentation, µ-Slides VI 0.1 (Ibidi GmbH, Graefelfing, Germany) were used, consisting of six equal units, each with a central channel and a reservoir above and below it (Figure 2). They were filled with collagen (PureCol^®^ Type I Bovine Collagen Solution; 3 mg/mL, Advanced Biomatrix, San Diego, CA, USA)-embedded cells supplemented with LAs and fluorescent dyes as displayed in Appendix A. Subsequently, samples were stored for a maximum of 1.5 h in an incubator at 37 °C and 5% CO_2_ under humid conditions until observation with the microscope.

#### 2.5.3. Migration and NETosis Assay after Hypotonic Lysis and Density Gradient Centrifugation

For migration and NETosis analysis after hypotonic lysis or density gradient centrifugation, 3D-µ-Slides were used. They were filled with collagen-embedded cells, LAs and fMLP as previously described by our department [25].

#### 2.5.4. Microscopy Setting

A Leica DFC9000 GT camera and a Leica Dmi8 microscope (both Leica Microsystems GmbH, Wetzlar, Germany) equipped with a climate chamber (Ibidi GmbH, Graefelfing, Germany) were used to observe the slides such that the slides were kept constant at 37 °C and 5% CO_2_ throughout the observation period. Using the LASX software program (version 3.4.2.18368, Leica Microsystems GmbH, Wetzlar, Germany), 1–10 images per channel and color setting were taken every 30 s for the 3D-µ-slides and every 5 or 10 min for the µ-Slides VI 0.1 over a period of up to 22 h. Four different color settings were chosen, one for phase contrast and one for each fluorescence used.

### 2.6. Flow Cytometry 

After gelafundin sedimentation, the cell samples were incubated with or without lidocaine, bupivacaine and levobupivacaine. After 0.5 h, 3 h, 6 h and 12 h, an oxidative burst and an antigen analysis for CD11b, CD62L and CD66b were performed, which has been described in detail by Kupke et al. and Trabold et al. [26,31]. Upon completion of the incubation period with the LAs, the burst was performed without stimulation and with stimulation by fMLP (10 µM, Sigma Aldrich, Steinheim, Germany) in combination with tumor necrosis factor alpha (TNF-α; 1 µg/mL, Thermo Fisher Scientific, Agawam, MA, USA), or with phorbol-12-myristate-13-acetate (PMA; 10 µM, Sigma Aldrich, Steinheim, Germany). ROS production was detected with DHR, which is converted to fluorescent rhodamine 123 by oxidation through reactive oxygen species. We used PE-conjugated anti-CD11b (ICRF44, BioLegend, San Diego, CA, USA), FITC-conjugated anti-CD62L (DREG-65, BioLegend, San Diego, CA, USA) and APC-conjugated anti-CD66b (G10F5, BioLegend, San Diego, CA, USA) to visualize the antigens. For detection, we used a FACS Calibur™ flow cytometer (BD corporate, Franklin Lakes, NJ, USA) and CellQuest Pro software™ (version 5.2, BD corporate, Franklin Lakes, NJ, USA).

### 2.7. Evaluation and Statistical Analysis

Imaris^®^ Microscopy Imaging Analysis Software (version 9.0.2, Bitplane, Zurich, Switzerland), Phoenix 64 NLME™ software (Build 8.1.0, Certara Inc., Princeton, NJ, USA), FlowJo™ (V10.0.7, BD corporate, Franklin Lakes, NJ, USA), Excel (Microsoft Corporation, Redmond, WA, USA), and the IBM^®^ SPSS^®^ statistics software program (version 29.0.0.0, IBM, Armonk, NY, USA) were used for data analysis. Tracks were included with a TD > 900 s and TL > 25 µm and a minimum number of 50 per set.

For statistical analysis, the Gaussian distribution was tested using the Kolmogorov–Smirnov test. Depending on the distribution, a Kruskal–Wallis test with a Bonferroni correction as the post hoc test or an ANOVA with a Levene and a Games–Howell test for more than two groups and a Mann–Whitney U test or T test for independent samples with a Levene test for two groups were performed. To test the correlation of two nominal scaled variables, we used a Fisher’s exact test. *p*-values less than 0.05 were considered statistically significant.

## 3. Results

In this first part, the results of PMN functions after gelafundin sedimentation are presented.

### 3.1. Antigen Expression—Flow Cytometry

The expression of CD62L shows a time-dependent decrease for all modalities, which is significant between 0.5 h and 12 h for control (*p* = 0.013), low concentrations of bupivacaine (*p* = 0.013) and both concentrations of levobupivacaine (low: *p* = 0.013, high: *p* = 0.013). All LAs at all concentrations except lidocaine at high concentration, the initial expression is higher than the control. 

The expression of CD11b and CD66b is lower than the control for all modalities at all points in time, being significant for high concentrations of lidocaine at 3 h for both antigens with *p* = 0.042 and at 12 h for CD11b with *p* = 0.013 (Table 2).

### 3.2. Viability and ROS Production—Flow Cytometry

With increasing time, the percentage of dead cells increases with and without stimulation. Higher percentages of dead cells occur with higher concentrations of LAs. Differences were significant for high concentrations of lidocaine at 12 h compared with control (without stimulation: *p* = 0.027, with fMLP: *p* = 0.037, with PMA: *p* = 0.033). 

Without stimulation, the ROS production measured with flow cytometry was low, with values from 2 up to 18. With stimulation through fMLP, low concentrations of bupivacaine and both concentration groups of levobupivacaine reached elevated levels at 3 h. With stimulation through PMA, maximal ROS production of the control was reached at 3 h. At this point, the ROS levels with LA are lower than the control; afterwards, a decrease in all modalities and the control occurs. At 12 h, the values with LA range between 2 and 13 and the control value is 155 (Table 3).

Table 4 shows in how many cases ROS production was measurable with live cell imaging in absolute and relative values. Without Las, the percentage of cases with measurable ROS production was higher than with the LA bupivacaine, levobupivacaine or lidocaine (Table 4). A statistical correlation between LA and measurability of ROS production exists (*p* < 0.001).

### 3.3. Migration—Live Cell Imaging

The migration results are shown separated by LAs.

#### 3.3.1. Bupivacaine - Migration

**Figure 3:**
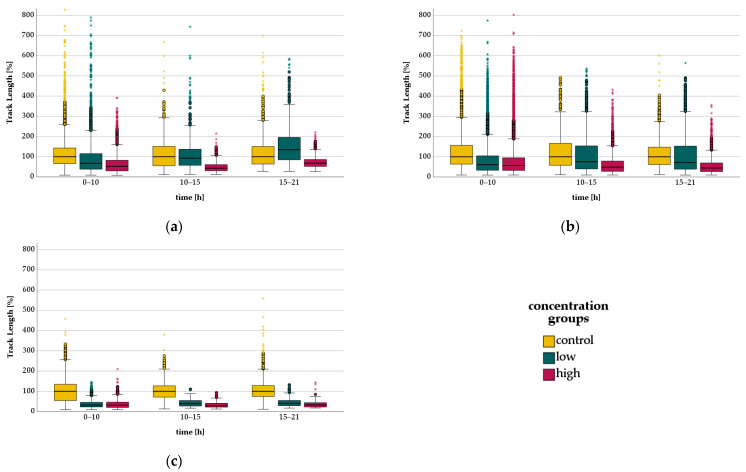
TLs of PMNs treated with (**a**) bupivacaine, (**b**) levobupivacaine and (**c**) lidocaine in all three periods arranged by concentration group. The time periods are divided into 0–10 h, 10–15 h and 15–21 h. Data are presented as boxplots. Not all outliers (circles) and extreme values (asterisks) are displayed.

TL showed a significant dose-dependent decrease in the first period. In the second and third period, there was a significantly increased TL in the low-concentration group compared to the control, while there was a significant decrease in the high-concentration group (Table 5). rTL showed a significant decrease in the first and second period, being dose-dependent in the second period. In the third period, a significantly increased rTL occurred in the low-concentration group compared to the control, while in the high-concentration group a significant decrease appeared (Figure 3).

TDX was significantly decreased in the first period in both concentration groups compared with the control, with a greater decrease at low concentrations than at high concentrations. rTDX showed a significant dose-dependent decrease in the first period compared with control (Table 6).

#### 3.3.2. Levobupivacaine-Migration

TL and rTL showed a dose-dependent reduction for all three time periods, being significant when compared to control (Figure 3 and Table 5). 

TDX and rTDX showed a significant reduction compared to the control. TDX and rTDX have a greater reduction at low than at high concentrations of levobupivacaine in the first period (Table 6).

#### 3.3.3. Lidocaine-Migration

TL and rTL showed a dose-dependent reduction in all three time periods, which was significant compared with control (Figure 3 and Table 5). 

TDX is significantly dose-dependently reduced compared to control in the first time period. rTDX showed a significant dose-dependent decrease in the first period (Table 6).

### 3.4. NETosis—Live Cell Imaging

**Figure 4:**
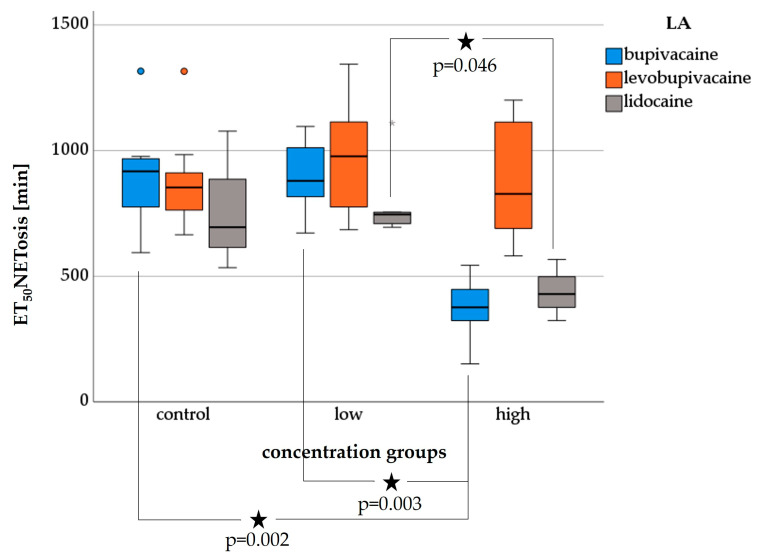
Impact of bupivacaine, levobupivacaine and lidocaine on ET_50_NETosis after gelafundin sedimentation presented as boxplots. Significant differences are indicated by asterisks (★) and the corresponding *p*-values. Outliers (circles) and extreme values (asterisks (*)) are displayed.

In the following section, the results regarding the NETosis of PMNs isolated by gelafundin sedimentation are presented (Figure 4).

#### 3.4.1. Bupivacaine-NETosis

ET_50_NETosis showed a dose-dependent reduction. PMNs treated with low concentrations had a median ET_50_NETosis of 880 min and PMNs treated with high concentrations had a median of 376 min, while the median of the control group was 918 min. The decrease in the high-concentration group was significant compared to the control (*p* = 0.002) and to the low-concentration group (*p* = 0.003).

#### 3.4.2. Levobupivacaine-NETosis

PMNs treated with levobupivacaine showed a slightly increased ET_50_NETosis for low concentrations with a median of 977 min compared with control (median 854 min), whereas the ET_50_NETosis for high concentrations decreased to a median of 828 min.

#### 3.4.3. Lidocaine-NETosis

PMNs treated with lidocaine showed a slightly increased median for ET_50_NETosis at low concentrations of 746 min compared to control (median 695 min), while the ET_50_NETosis decreased to a median of 429 min at high concentrations. The decrease at high concentrations is significant compared to that at low concentrations.

### 3.5. Migration and NETosis with Autologous Serum and FCS after Gelafundin Isolation—Live Cell Imaging

The control had a median TL of 169 µm with autologous serum and 146 µm with FCS in the first period. The control had a median TDX of 15.8 µm with autologous serum and 5.35 µm with FCS in the first period. The control had a median ET_50_NETosis of 907 min with autologous serum and 974 min with FCS. No significant differences were detected for TL in the first period (*p* = 0.819) and NETosis (*p* = 0.854) between autologous serum and FCS. TDX significantly differed between PMNs treated with autologous serum and FCS in the first period (*p* < 0.001).

### 3.6. Comparison of Isolation Methods—Live Cell Imaging

In this second part, the results of the PMN functions according to the three isolation methods are presented.

#### 3.6.1. Migration

The TL of the controls from 60 to 90 min shows a significant difference between the three isolation techniques (both *p* = 0.001). PMNs isolated with hypotonic lysis had a median of 184 µm, with density gradient centrifugation of 80 µm and with gelafundin sedimentation of 95 µm. Migration could be detected with hypotonic lysis up to 2.5 h, with density gradient centrifugation up to 3.7 h, and with gelafundin sedimentation, migration was even detectable up to 21.5 h.

#### 3.6.2. ET_50_NETosis

The ET_50_NETosis of the controls shows a significant difference between the three isolation techniques. PMNs isolated with hypotonic lysis have a median ET_50_NETosis of 287 min, with density gradient centrifugation of 424 min and with gelafundin sedimentation of 875 min. PMNs isolated with gelafundin sedimentation differ from the rest with *p* < 0.001, and PMNs isolated with hypotonic lysis or density gradient centrifugation differ from each other with *p* = 0.034.

#### 3.6.3. EC_50_NETosis

**Figure 5:**
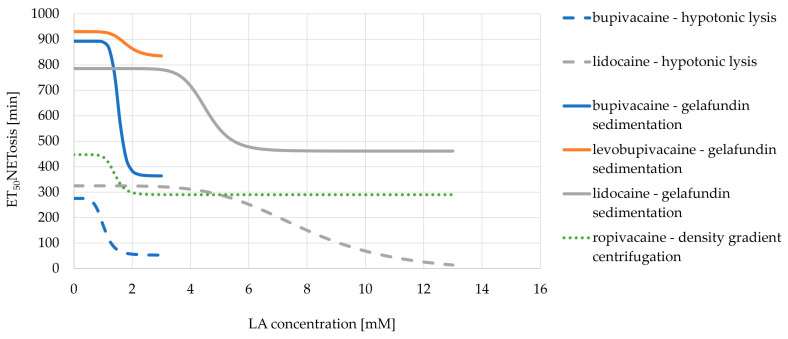
Effects of LAs on the ET_50_NETosis separated by isolation method. The different LAs are displayed via colors. The isolation methods are displayed via line type.

When ET_50_NETosis values are plotted against the concentration, a sigmoidal curve can be generated for each LA, modeling the progression of ET_50_NETosis and displaying a calculated individual EC_50_NETosis value for each LA, which represents the concentration to reach the half-maximal reduction in ET_50_NETosis for each LA (Figure 5).

The reduction is much greater for bupivacaine than for levobupivacaine. However, they have similar EC_50_NETosis values. The calculated EC_50_NETosis value for bupivacaine with gelafundin sedimentation is 1.53 mM and with hypotonic lysis it is 1.02 mM. For levobupivacaine with gelafundin sedimentation, it is 1.79 mM. For lidocaine with gelafundin sedimentation, it is 4.54 mM and with hypotonic lysis it is 7.91 mM. For ropivacaine with density gradient centrifugation, EC_50_NETosis is 1.41 mM.

## 4. Discussion

This study investigates the influences of different LAs at different concentrations on selected functions of PMNs obtained with different isolation methods. 

The concentrations of LAs were chosen based on previous studies and according to clinically applied dosages [23,25,32]. Bupivacaine and levobupivacaine were used for infiltration at initial maximal concentrations of 15.4 mmol/L. Because of the immediate onset of diffusion in the tissue, we chose a much lower concentration for the study. For lidocaine, this initial maximal concentration is 69.2 mmol/L, and for ropivacaine, it is 7.29 mmol/L. For ropivacaine, additional concentrations above clinical use were tested. 

It is known that CD62L levels decrease in response to neutrophil stimulation [32,33]. 

Our results show that PMNs treated with and without LAs are stimulated over time as CD62L expression decreases. Except for lidocaine at high concentrations, all PMNs treated with local anesthetics are initially less stimulated and experience delayed stimulation until they are even more activated than the control at later observation times. As CD11b and CD66b levels rise on the cell surface upon PMN activation [32,33,34,35], there seems to be an activation of the control within 0.5 and 3 h after isolation. PMNs treated with LAs remain consistently at low expression levels and do not appear to undergo activation. Similar results were found for CD11b in some studies [24,36], but contrasting results also exist for all three tested surface antigens [25]. 

Dihydrorhodamine 123 (DHR), which is converted to the fluorescent dye rhodamine 123 by oxidation, is used to detect ROS [37,38]. In principle, rhodamine 123 can stain mitochondria, but as seen in our control group, without LA and without oxidative burst activation by fMLP or PMA, only baseline low fluorescence is detectable (Table 3) [39,40]. The time course of ROS production as detected by flow cytometry and the cell-wide staining of the generated ROS as detected by live cell imaging additionally indicate the absence of noteworthy mitochondrial ROS production. The LAs lidocaine, bupivacaine and levobupivacaine do not induce ROS production without stimulus. Moreover, they suppress ROS production stimulated by fMLP or PMA. This effect is consistent with the results of other studies on lidocaine and bupivacaine [22,23,24]. The impact of levobupivacaine and bupivacaine is similar with respect to the modulation of ROS production. At low concentrations, they do not suppress ROS production as much as at high concentrations. For lidocaine, a strong suppression of ROS production is seen at both low and high concentrations. The results found by flow cytometry are consistent with live cell imaging results. Here, ROS production is completely suppressed by lidocaine without stimulation, whereas it is only partially suppressed by levobupivacaine and bupivacaine.

The addition of LA to a single reservoir for migration analysis after gelafundin sedimentation dilutes the LA concentration over time by diffusion throughout the volume of the unit of a 3D-µ-slide. This may account for the development of TL and rTL of PMNs treated with low concentrations of bupivacaine or levobupivacaine assuming that there is a threshold concentration for a measurable effect. The low-concentration group for bupivacaine and levobupivacaine reaches the required concentration for a measurable effect up to a certain time point. At all time points thereafter, the effect decreases again such that TL is higher in the second period, with low concentrations of bupivacaine and levobupivacaine, than in the first period. PMNs treated with low concentrations of bupivacaine sometimes achieve even higher values for TL than the control. For the high-concentration groups of bupivacaine and levobupivacaine and for both concentration groups of lidocaine, even after dilution, the minimum concentration for an effect is reached and exceeded. This might explain why there is no increase in the migration parameters at later times for this groups. Kolle et al. reports similar effects for lidocaine at concentrations lower than we applied [25].

The direction of chemotaxis towards an attractant (rTDX) is inhibited by the tested LAs significantly more than the route of chemotaxis (rTL). This suggests that direction finding during chemotaxis is significantly more affected than chemokinesis itself. The signaling pathways for chemotaxis contain many steps that may be differentially affected by LAs. Approaching points could be, for example, the receptors or the localization of PIP_3_. G-protein-coupled fMLP and chemokine receptors, such as FPR1 and FPR2 or CXCR1, are important at the beginning of signal transduction [41]. In the further course of the signaling cascade, PIK3 converts PIP_2_ to PIP_3_, which accumulates in specific areas of the cell on the side where the chemoattractant has bound to the receptors. This restricted localization is critical for directional migration along the chemotactic gradient [42,43]. This restricted localization is maintained by PTEN in *Dictyostelium* and by SHIP1 in *mice* [44,45], which may be of interest for further studies to better understand the molecular action of LAs upon PMNs. 

With bupivacaine and levobupivacaine, the percentage of dead cells increases only slightly, whereas with lidocaine the percentage increases very strongly. In conjunction with the results of NETosis, lidocaine and bupivacaine cause cells to become NETotic at an earlier time point, both after gelafundin sedimentation and after hypotonic lysis. Ropivacaine and levobupivacaine, on the other hand, barely trigger an earlier time to half-maximal NETosis compared to PMNs without LA.

The PMNs under the influence of the tested LAs show no or later activation in terms of antigen expression, as well as limitation of chemotaxis and ROS production, with lidocaine showing a much stronger effect than bupivacaine and levobupivacaine. Similar results are also seen in the percentage of dead PMNs after incubation with the LAs. Differences between the LAs, especially between racemic bupivacaine and levobupivacaine, are seen in NETosis, which is mainly influenced by lidocaine and bupivacaine, but not by ropivacaine and levobupivacaine. In other studies, lidocaine has also been shown to have an inhibitory impact on cytokine secretion [46,47]. These altered functions of PMNs suggest that their pathogen control functions are affected in the short term. Premature NETosis, even in the absence of a pathogen, leads to unnecessary inflammation at the wrong sites. This is consistent with the occurrence of increased NETosis makers in autoimmune diseases [48,49,50,51,52]. In addition, PMNs are prevented from reaching the site of pathogen entry, if present. As an important component of the innate immune system, PMNs contribute to combating pathogens through their various functions, such as ROS production and NETosis, which we have examined, and phagocytosis [1,3]. In addition, they also play an important role in the immune response through their interaction with other immune cells and components, including IgG and the complement system [1]. The impact of impaired PMN functions is particularly comprehensible in patients with chronic granulomatous disease (CGD). A decreased activation and suppressed ROS in the presence of a stimulus leads to an inadequate response to pathogens and pathological situations as in CGD patients, who are therefore more susceptible to infections [53]. Infections are also well-known complications of surgical procedures. 

As touched upon above for migration, different signaling pathways play important roles in the PMN functions studied [1,41]. Since LAs have varying influences on the different parameters, this suggests that different signaling pathways are elementary for the different functions. For NETosis, after triggering receptors such as GPCRs, Fcy, TLR4, and complement receptors, the influence of calcium from the endoplasmic reticulum into the intracellular space seems to be an important component of signal conduction [54]. The influence of ROS on NETosis is also discussed, which seems to depend on the stimulus. In PMA-induced NETosis, ROS production seems to play an important part; in contrast, the bacterial toxin nigericin, which acts as a potassium ionophore, induces NETosis without inducing ROS production [55].

In one isolation technique, we used gelafundin for isolation to accelerate the segregation of erythrocytes and leukocytes. We discovered the major difference between the isolation methods in the use of FCS or autologous serum, as well as in the centrifugation steps applied. For hypotonic stimuli, their influence on sodium currents in *rat* trigeminal neurons was found to be largely reversible after the washout of hypotonicity; furthermore, lidocaine has been reported to have an effect on PMNs that is not associated with the inhibition of sodium channels [47,56]. Because there were no differences between the TL and ET_50_NETosis of the two groups in the experiments using gelafundin sedimentation with FCS or autologous serum, it is reasonable to assume that the different results of the isolation methods are due to centrifugation [30,57]. Of particular interest is the significantly lower time for ET_50_NEtosis and the shorter migration span observed. This suggests that PMNs are already limited in function by hypotonic lysis and density gradient centrifugation prior to the addition of LAs by the isolation procedure. After gelafundin sedimentation, PMNs appeared to have a behavior more in line with the survival time in tissue of 1–4 days [1,58]. This is consistent with Rimboeck et al., who presented data on the viability of PMNs with gelafundin sedimentation for up to 150 h [57]. The ET_50_NETosis values shift strongly in parallel by centrifugation. In contrast, the EC_50_NETosis values of the different LAs change moderately and are not shifted in the same direction by centrifugation. The EC_50_NETosis for lidocaine increases slightly and the EC_50_NETosis for bupivacaine decreases slightly as the result of centrifugation. Therefore, we conclude that centrifugation does not lead to a disturbance of the signal transduction triggered by LAs.

Gelafundin sedimentation seems to be the most recent isolation technique to obtain leukocyte-rich plasma, including PMNs, as natively as possible. Since we could not ensure high purity of the cells via the isolation methods, the subsequent usage of a migration assay through a collagen I gel or the usage of flow cytometry in combination with the detection of CD62L, CD11b and CD66b allows for the observation of pure PMNs. To ensure comparability of data between samples despite possible differences in the yield of isolation methods, parameters were chosen that are independent of cell numbers. All results mentioned above were found in vitro and would need to be supported by clinical trials, whereas LAs would need to be compared in terms of their effect on the immune system and in particular on the functions of PMNs. There are some studies that analyze this for single LAs such as ropivacaine [59] and lidocaine [60]. For lidocaine, there is also a study that refers not to PMNs but only to natural killer cells and lymphocytes [61].

## 5. Conclusions

The tested LAs have varying influences on the investigated PMN functions. Of particular interest are the different effects of racemic bupivacaine and levobupivacaine on NETosis, as levobupivacaine has little accelerating effect, but bupivacaine shows a large accelerating effect on NETosis onset. This suggests that the careful selection of LAs has a short-term impact on in vitro PMNs. We also could verify that isolation methods affect the behavior of PMNs, with gelafundin sedimentation being particularly gentle. 

## Figures and Tables

**Table 1:** Methods, PMN functions and observed parameters.

Method	PMN Function	Parameter
Live Cell Imaging	Migration	Track length (TL)
		Track displacement along the chemotactic gradient (TDX)
		Track duration (TD)
		Observation time
	NETosis	Time for half-maximal NETosis (ET_50_NETosis)
		Concentration to reach half-maximal reduction of ET_50_NETosis for each LA (EC_50_NETosis)
	ROS production	Percentage of detectable cases
Flow cytometry	Viability	Percentage of dead cells
	ROS production	Conversion to rhodamine 123
	Activation	Expression of CD62L, CD11b, CD66b

**Table 2:** Median ± standard deviation intensity of the expression of antigens CD62L, CD11b and CD66b arranged by LA, concentration group and incubation time. Number of measurements n = 3.

LA	Concentration Group	Incubation Time [h]	CD62L	CD11b [×10^2^]	CD66b [×10^2^]
Control	Control	0.5	63.8 ± 7.81	11.9 ± 6.42	3.72 ± 1.57
		3	27.4 ± 2.43	23.3 ± 0.89	13.7 ± 2.35
		6	24.6 ± 0.473	20.9 ± 1.64	12.9 ± 2.36
		12	19.1 ± 1.97	17.9 ± 2.20	11.6 ± 2.14
Bupivacaine	Low	0.5	149 ± 9.45	2.55 ± 1.68	2.09 ± 0.19
		3	82.7 ± 12.1	6.55 ± 1.47	3.11 ± 0.87
		6	41.0 ± 4.29	8.74 ± 4.75	4.10 ± 0.32
		12	10.4 ± 5.20	3.68 ± 4.53	2.04 ± 0.79
	High	0.5	132 ± 34.8	3.68 ± 0.65	2.07 ± 0.28
		3	85.1 ± 52.7	2.39 ± 1.61	2.53 ± 0.84
		6	40.0 ± 21.3	2.48 ± 2.67	2.23 ± 0.52
		12	8.7 ± 1.5	3.82 ± 0.31	2.05 ± 0.16
Levobupivacaine	Low	0.5	137 ± 14.6	1.83 ± 1.63	2.05 ± 0.25
		3	94.7 ± 17.8	0.784 ± 2.98	2.53 ± 0.61
		6	48.7 ± 13.1	4.26 ± 7.34	2.59 ± 1.76
		12	9.0 ± 6.1	4.22 ± 4.87	2.17 ± 0.92
	High	0.5	137 ± 19.2	3.46 ± 1.60	2.15 ± 0.79
		3	97.3 ± 28.3	7.64 ± 2.41	3.13 ± 0.53
		6	42.6 ± 2.85	4.14 ± 8.23	2.11 ± 2.04
		12	8.8 ± 6.0	4.37 ± 4.97	2.25 ± 1.16
Lidocaine	Low	0.5	121 ± 23.7	2.11 ± 1.05	1.88 ± 0.28
		3	22.1 ± 37.1	4.10 ± 1.37	2.37 ± 0.54
		6	9.1 ± 5.5	3.53 ± 0.12	2.11 ± 0.42
		12	8.1 ± 1.1	3.62 ± 0.13	2.41 ± 0.19
	High	0.5	25.9 ± 18.1	3.05 ± 1.40	2.37 ± 0.25
		3	6.7 ± 1.4	0.573 ± 1.53 *	1.95 ± 0.39 *
		6	7.6 ± 1.2	2.55 ± 0.63	2.13 ± 0.31
		12	9.7 ± 1.1	2.69 ± 1.26 *	5.00 ± 2.73

*: significant changes compared to the control group.

**Table 3:** Median ± standard deviation of dead cells and ROS without stimulation and after stimulation with fMLP and PMA arranged by LA, concentration group and incubation time. Measurement was performed with flow cytometry. Number of measurements n = 3.

LA	Concentration Group	Incubation Time [h]	Dead [%]			Rhodamin 123		
			No Activation	FMLP	PMA	No Activation	FMLP	PMA
Control	Control	0.5	0.52 ± 0.17	0.98 ± 0.66	0.81 ± 0.26	2 ± 3	46 ± 66	437 ± 542
		3	0.79 ± 0.22	0.44 ± 0.61	0.81 ± 3.91	2 ± 4	13 ± 20	1610 ± 1070
		6	1.00 ± 0.36	0.56 ± 0.86	0.67 ± 0.85	13 ± 8	34 ± 22	723 ± 448
		12	1.59 ± 0.06	1.50 ± 0.66	2.01 ± 0.85	3 ± 13	16 ± 26	155 ± 181
Bupivacaine	Low	0.5	0.46 ± 0.09	0.35 ± 0.08	0.57 ± 0.63	2 ± 4	31 ± 59	359 ± 930
		3	0.29 ± 0.10	0.28 ± 0.08	0.38 ± 0.70	9 ± 5	106 ± 48	1100 ± 913
		6	0.69 ± 0.17	0.59 ± 0.29	0.89 ± 0.80	11 ± 7	47 ± 20	1030 ± 717
		12	1.92 ± 1.40	1.50 ± 1.78	2.12 ± 2.06	7 ± 9	1 ± 10	2 ± 15
	High	0.5	1.01 ± 0.50	0.60 ± 0.30	0.69 ± 1.87	2 ± 4	13 ± 37	389 ± 891
		3	1.16 ± 1.69	1.52 ± 1.92	0.65 ± 3.66	11 ± 6	22 ± 44	751 ± 588
		6	1.58 ± 3.12	1.75 ± 3.77	1.75 ± 3.67	9 ± 8	12 ± 6	246 ± 227
		12	6.67 ± 21.8	7.18 ± 21.6	7.58 ± 20.2	10 ± 7	10 ± 7	10 ± 7
Levobupivacaine	Low	0.5	0.52 ± 0.38	0.67 ± 0.31	1.39 ± 0.89	2 ± 4	23 ± 46	441 ± 891
		3	0.23 ± 0.15	0.39 ± 0.22	0.30 ± 1.82	10 ± 5	106 ± 46	1260 ± 1190
		6	0.64 ± 0.27	0.62 ± 0.11	0.85 ± 0.77	9 ± 8	20 ± 27	538 ± 609
		12	3.01 ± 1.58	3.10 ± 0.66	3.07 ± 0.95	11 ± 9	12 ± 9	12 ± 28
	High	0.5	0.86 ± 0.49	0.42 ±0.23	1.05 ± 0.73	3 ± 4	12 ± 34	594 ± 828
		3	0.57 ± 0.56	0.79 ± 0.23	2.11 ± 2.66	10 ± 5	57 ± 27	1240 ± 1140
		6	0.79 ± 0.13	0.78 ± 0.08	0.83 ± 2.80	9 ± 8	12 ± 36	368 ± 714
		12	5.92 ± 7.23	4.69 ± 2.97	5.80 ± 7.41	12 ± 8	12 ± 9	13 ± 29
Lidocaine	Low	0.5	1.54 ± 0.81	1.26 ± 1.70	2.02 ± 1.21	2 ± 4	17 ± 23	331 ± 763
		3	7.27 ± 4.37	6.96 ± 4.95	7.49 ± 4.46	18 ± 10	28 ± 14	947 ± 666
		6	8.49 ± 10.2	7.36 ± 9.64	8.84 ± 8.55	11 ± 7	12 ± 5	28 ± 77
		12	23.8 ± 33.1	27.6 ± 32.4	33.1 ± 26.7	2 ± 10	2 ± 9	9 ± 9
	High	0.5	5.68 ± 0.54	7.03 ± 2.82	8.26 ± 2.43	3 ± 4	6 ± 23	466 ± 827
		3	10.9 ± 2.40	10.4 ± 1.66	11.6 ± 2.44	18 ± 9	19 ± 10	19 ± 148
		6	25.0 ± 22.2	21.6 ± 24.5	20.4 ± 21.5	12 ± 7	12 ± 6	13 ± 4
		12	74.2 ± 10.2 *	68.7 ± 13.4 *	67.6 ± 22.6 *	2 ± 12	1 ± 11	2 ± 11

*: significant changes compared to the control group.

**Table 4:** Frequency of measurable ROS production with live cell imaging. The low-concentration group and high-concentration group of each LA are included together. The percentage values refer to the corresponding overall percentages of the control/LA.

	Control	Bupivacaine	Levobupivacaine	Lidocaine
Not measurable	5	15	12	10
Not measurable [%]	19.2	68.2	60.0	100
Measurable	21	7	8	0
Measurable [%]	80.8	31.8	40.0	0.0
Overall	26	22	20	10

**Table 5:** Median of TL and relative TL (rTL) arranged by LA, observed period and LA concentration group.

LA	Time [h]	Concentration Group	TL [µm/30 min]	rTL [%]
Bupivacaine	0–10	Control	135	100
		Low	83.1 *	67.6 *
		High	77.1 *	52.1 *
	10–15	Control	105	100
		Low	136 *	92.8 *
		High	66.8 *	43.1 *
	15–21	Control	81.1	100
		Low	112 *	135 *
		High	56.7 *	68.0 *
Levobupivacaine	0–10	Control	100	100
		Low	82.4 *	61.8 *
		High	77.5 *	56.7 *
	10–15	Control	126	100
		Low	116 *	75.5 *
		High	73.2 *	48.8 *
	15–21	Control	145	100
		Low	108 *	72.1 *
		High	67.7 *	44.8 *
Lidocaine	0–10	Control	173	100
		Low	63.8 *	32.3 *
		High	53.4 *	31.6 *
	10–15	Control	199	100
		Low	56.9 *	39.5 *
		High	50.0 *	28.9 *
	15–21	Control	207	100
		Low	45.2 *	40.8 *
		High	39.2 *	33.4 *

*: significant changes compared to the control group.

**Table 6:** Median of TDX and relative TDX (rTDX) arranged by LA and LA concentration group in the first period.

LA	Time [h]	Concentration Group	TDX [µm/30 min]	rTDX [%]
Bupivacaine	0–10	Control	27.5	100
		Low	1.6 *	5.0 *
		High	2.0 *	5.6 *
Levobupivacaine	0–10	Control	11.2	100
		Low	1.2 *	5.1 *
		High	2.1 *	8.9 *
Lidocaine	0–10	Control	8.3	100
		Low	0.9 *	3.9 *
		High	0.2 *	0.6 *

*: significant changes compared to the control group.

## Data Availability

The data presented in this work are available on request from the corresponding author.

## Appendix A

**Table A1:** Contents of the different compartments of the slides used.

Assay	Left/Upper Reservoir	Channel	Right/Lower Reservoir
Migration assay after gelafundin sedimentation	fMLP with LA in RPMI and autologous serum	Medium (17%) *	Cells in autologous serum
		Collagen (50%)	
		RPMI (33%)	
NETosis and ROS assay after gelafundin sedimentation	Medium (13%) * Collagen (50%) Cells (13%) LA (25%)	Medium (13%) * Collagen (50%) Cells (13%) LA (25%)	Medium (13%) * Collagen (50%) Cells (13%) LA (25%)
Migration and NETosis assay after hypotonic lysis and density gradient centrifugation	fMLP with LA in RPMI and FCS	Medium (17%) *	LA in RPMI and FCS
		Collagen (50%)	
		Cells (33%)	

*: medium: minimal essential medium Eagle (MEME × 10; Sigma-Aldrich) (6.7%), aqua dest. (6.7%). sodium bicarbonate solution (NaHCO_3_; Sigma-Aldrich) (3.3%).

## References

[1] Mayadas T.N., Cullere X., Lowell C.A. (2014). The multifaceted functions of neutrophils. Annu. Rev. Pathol..

[2] Vorobjeva N.V., Chernyak B.V. (2020). NETosis: Molecular Mechanisms, Role in Physiology and Pathology. Biochemistry.

[3] Borregaard N. (2010). Neutrophils, from marrow to microbes. Immunity.

[4] Borregaard N., Kjeldsen L., Lollike K., Sengeløv H. (1995). Granules and secretory vesicles of the *human* neutrophil. Clin. Exp. Immunol..

[5] Fuchs T.A., Abed U., Goosmann C., Hurwitz R., Schulze I., Wahn V., Weinrauch Y., Brinkmann V., Zychlinsky A. (2007). Novel cell death program leads to neutrophil extracellular traps. J. Cell Biol..

[6] Brinkmann V., Reichard U., Goosmann C., Fauler B., Uhlemann Y., Weiss D.S., Weinrauch Y., Zychlinsky A. (2004). Neutrophil extracellular traps kill bacteria. Science.

[7] Zuo Y., Yalavarthi S., Shi H., Gockman K., Zuo M., Madison J.A., Blair C., Weber A., Barnes B.J., Egeblad M. (2020). Neutrophil extracellular traps in COVID-19. JCI Insight.

[8] Ronchetti L., Terrenato I., Ferretti M., Corrado G., Goeman F., Donzelli S., Mandoj C., Merola R., Zampa A., Carosi M. (2022). Circulating cell free DNA and citrullinated histone H3 as useful biomarkers of NETosis in endometrial cancer. J. Exp. Clin. Cancer Res. CR.

[9] Telerman A., Granot G., Leibovitch C., Yarchovsky-Dolberg O., Shacham-Abulafia A., Partouche S., Yeshurun M., Ellis M.H., Raanani P., Wolach O. (2021). Neutrophil Extracellular Traps Are Increased in Chronic Myeloid Leukemia and Are Differentially Affected by Tyrosine Kinase Inhibitors. Cancers.

[10] Stehr A.M., Wang G., Demmler R., Stemmler M.P., Krug J., Tripal P., Schmid B., Geppert C.I., Hartmann A., Muñoz L.E. (2022). Neutrophil extracellular traps drive epithelial-mesenchymal transition of *human* colon cancer. J. Pathol..

[11] Li Y., Yang Y., Gan T., Zhou J., Hu F., Hao N., Yuan B., Chen Y., Zhang M. (2019). Extracellular RNAs from lung cancer cells activate epithelial cells and induce neutrophil extracellular traps. Int. J. Oncol..

[12] Kaltenmeier C.T., Yazdani H., van der Windt D., Molinari M., Geller D., Tsung A., Tohme S. (2021). Neutrophil extracellular traps as a novel biomarker to predict recurrence-free and overall survival in patients with primary hepatic malignancies. HPB Off. J. Int. Hepato Pancreato Biliary Assoc..

[13] Lee W., Ko S.Y., Mohamed M.S., Kenny H.A., Lengyel E., Naora H. (2019). Neutrophils facilitate ovarian cancer premetastatic niche formation in the omentum. J. Exp. Med..

[14] Yang L., Liu Q., Zhang X., Liu X., Zhou B., Chen J., Huang D., Li J., Li H., Chen F. (2020). DNA of neutrophil extracellular traps promotes cancer metastasis via CCDC25. Nature.

[15] Kwak S.-B., Kim S.J., Kim J., Kang Y.-L., Ko C.W., Kim I., Park J.-W. (2022). Tumor regionalization after surgery: Roles of the tumor microenvironment and neutrophil extracellular traps. Exp. Mol. Med..

[16] Paunel-Görgülü A., Wacker M., El Aita M., Hassan S., Schlachtenberger G., Deppe A., Choi Y.-H., Kuhn E., Mehler T.O., Wahlers T. (2017). cfDNA correlates with endothelial damage after cardiac surgery with prolonged cardiopulmonary bypass and amplifies NETosis in an intracellular TLR9-independent manner. Sci. Rep..

[17] Becker D.E., Reed K.L. (2012). Local anesthetics: Review of pharmacological considerations. Anesth. Prog..

[18] Katzung B.G., Vanderah T.W. (2021). Basic et Clinical Pharmacology.

[19] Blumenthal S., Borgeat A., Pasch T., Reyes L., Booy C., Lambert M., Schimmer R.C., Beck-Schimmer B. (2006). Ropivacaine decreases inflammation in experimental endotoxin-induced lung injury. Anesthesiology.

[20] Finnerty D.T., Buggy D.J. (2020). A novel role for lidocaine in COVID-19 patients?. Br. J. Anaesth..

[21] Hollmann M.W., Durieux M.E. (2000). Local anesthetics and the inflammatory response: A new therapeutic indication?. Anesthesiology.

[22] Azuma Y., Shinohara M., Wang P.L., Suese Y., Yasuda H., Ohura K. (2000). Comparison of inhibitory effects of local anesthetics on immune functions of neutrophils. Int. J. Immunopharmacol..

[23] Hattori M., Dohi S., Nozaki M., Niwa M., Shimonaka H. (1997). The inhibitory effects of local anesthetics on superoxide generation of neutrophils correlate with their partition coefficients. Anesth. Analg..

[24] Kiefer R.-T., Ploppa A., Krueger W.A., Plank M., Nohé B., Haeberle H.A., Unertl K., Dieterich H.-J. (2003). Local anesthetics impair *human* granulocyte phagocytosis activity, oxidative burst, and CD11b expression in response to *Staphylococcus aureus*. Anesthesiology.

[25] Kolle G., Metterlein T., Gruber M., Seyfried T., Petermichl W., Pfaehler S.-M., Bitzinger D., Wittmann S., Bredthauer A. (2021). Potential Impact of Local Anesthetics Inducing Granulocyte Arrest and Altering Immune Functions on Perioperative Outcome. J. Inflamm. Res..

[26] Trabold B., Gruber M., Fröhlich D. (2007). Functional and phenotypic changes in polymorphonuclear neutrophils induced by catecholamines. Scand. Cardiovasc. J..

[27] Bitzinger D.I., Zausig Y.A., Paech C., Gruber M., Busse H., Sinner B., Graf B.M., Trabold B. (2013). Modulation of immune functions in polymorphonuclear neutrophils induced by physostigmine, but not neostigmine, independent of cholinergic neurons. Immunobiology.

[28] Hollmann M.W., Kurz K., Herroeder S., Struemper D., Hahnenkamp K., Berkelmans N.S., den Bakker C.G., Durieux M.E. (2003). The effects of S(-)-, R(+)-, and racemic bupivacaine on lysophosphatidate-induced priming of *human* neutrophils. Anesth. Analg..

[29] Welters I.D., Menzebach A., Langefeld T.W., Menzebach M., Hempelmann G. (2001). Inhibitory effects of S-(-) and R-(+) bupivacaine on neutrophil function. Acta Anaesthesiol. Scand..

[30] Hundhammer T., Gruber M., Wittmann S. (2022). Paralytic Impact of Centrifugation on *Human* Neutrophils. Biomedicines.

[31] Kupke L.S., Arndt S., Lenzer S., Metz S., Unger P., Zimmermann J.L., Bosserhoff A.-K., Gruber M., Karrer S. (2021). Cold Atmospheric Plasma Promotes the Immunoreactivity of Granulocytes In Vitro. Biomolecules.

[32] Ivetic A., Hoskins Green H.L., Hart S.J. (2019). L-selectin: A Major Regulator of Leukocyte Adhesion, Migration and Signaling. Front. Immunol..

[33] Kishimoto T.K., Jutila M.A., Berg E.L., Butcher E.C. (1989). Neutrophil Mac-1 and MEL-14 adhesion proteins inversely regulated by chemotactic factors. Science.

[34] Torsteinsdóttir I., Arvidson N.G., Hällgren R., Håkansson L. (1999). Enhanced expression of integrins and CD66b on peripheral blood neutrophils and eosinophils in patients with rheumatoid arthritis, and the effect of glucocorticoids. Scand. J. Immunol..

[35] Zhao L., Xu S., Fjaertoft G., Pauksen K., Håkansson L., Venge P. (2004). An enzyme-linked immunosorbent assay for *human* carcinoembryonic antigen-related cell adhesion molecule 8, a biological marker of granulocyte activities in vivo. J. Immunol. Methods.

[36] Ploppa A., Kiefer R.-T., Krueger W.A., Unertl K.E., Durieux M.E. (2008). Local anesthetics time-dependently inhibit *staphylococcus aureus* phagocytosis, oxidative burst and CD11b expression by *human* neutrophils. Reg. Anesth Pain Med..

[37] Henderson L.M., Chappell J.B. (1993). Dihydrorhodamine 123: A fluorescent probe for superoxide generation?. Eur. J. Biochem..

[38] Dupré-Crochet S., Erard M., Nüβe O. (2013). ROS production in phagocytes: Why, when, and where?. J. Leukoc. Biol..

[39] Panaro M.A., Mitolo V. (1999). Cellular responses to FMLP challenging: A mini-review. Immunopharmacol. Immunotoxicol..

[40] Chen L.B. (1988). Mitochondrial membrane potential in living cells. Annu. Rev. Cell Biol..

[41] Futosi K., Fodor S., Mócsai A. (2013). Neutrophil cell surface receptors and their intracellular signal transduction pathways. Int. Immunopharmacol..

[42] Smirnova T., Segall J.E. (2007). Amoeboid chemotaxis: Future challenges and opportunities. Cell Adhes. Migr..

[43] Xu J., Wang F., van Keymeulen A., Herzmark P., Straight A., Kelly K., Takuwa Y., Sugimoto N., Mitchison T., Bourne H.R. (2003). Divergent signals and cytoskeletal assemblies regulate self-organizing polarity in neutrophils. Cell.

[44] Bagorda A., Parent C.A. (2008). Eukaryotic chemotaxis at a glance. J. Cell Sci..

[45] Nishio M., Watanabe K., Sasaki J., Taya C., Takasuga S., Iizuka R., Balla T., Yamazaki M., Watanabe H., Itoh R. (2007). Control of cell polarity and motility by the PtdIns(3,4,5)P3 phosphatase SHIP1. Nat. Cell Biol..

[46] Ali Z.A., El-Mallakh R.S. (2020). Nebulized Lidocaine in COVID-19, An Hypothesis. Med. Hypotheses.

[47] Karnina R., Arif S.K., Hatta M., Bukhari A. (2021). Molecular mechanisms of lidocaine. Ann. Med. Surg..

[48] Sur C.C., Giaglis S., Walker U.A., Buser A., Hahn S., Hasler P. (2014). Enhanced neutrophil extracellular trap generation in rheumatoid arthritis: Analysis of underlying signal transduction pathways and potential diagnostic utility. Arthritis Res. Ther..

[49] Carmona-Rivera C., Zhao W., Yalavarthi S., Kaplan M.J. (2015). Neutrophil extracellular traps induce endothelial dysfunction in systemic lupus erythematosus through the activation of matrix metalloproteinase-2. Ann. Rheum. Dis..

[50] Garcia-Romo G.S., Caielli S., Vega B., Connolly J., Allantaz F., Xu Z., Punaro M., Baisch J., Guiducci C., Coffman R.L. (2011). Netting neutrophils are major inducers of type I IFN production in pediatric systemic lupus erythematosus. Sci. Transl. Med..

[51] Hakkim A., Fürnrohr B.G., Amann K., Laube B., Abed U.A., Brinkmann V., Herrmann M., Voll R.E., Zychlinsky A. (2010). Impairment of neutrophil extracellular trap degradation is associated with lupus nephritis. Proc. Natl. Acad. Sci. USA.

[52] Villanueva E., Yalavarthi S., Berthier C.C., Hodgin J.B., Khandpur R., Lin A.M., Rubin C.J., Zhao W., Olsen S.H., Klinker M. (2011). Netting neutrophils induce endothelial damage, infiltrate tissues, and expose immunostimulatory molecules in systemic lupus erythematosus. J. Immunol..

[53] Stasia M.J., Li X.J. (2008). Genetics and immunopathology of chronic granulomatous disease. Semin. Immunopathol..

[54] Thiam H.R., Wong S.L., Wagner D.D., Waterman C.M. (2020). Cellular Mechanisms of NETosis. Annu. Rev. Cell Dev. Biol..

[55] Kenny E.F., Herzig A., Krüger R., Muth A., Mondal S., Thompson P.R., Brinkmann V., von Bernuth H., Zychlinsky A. (2017). Diverse stimuli engage different neutrophil extracellular trap pathways. eLife.

[56] Chen L., Liu C., Liu L., Cao X. (2009). Changes in osmolality modulate voltage-gated sodium channels in trigeminal ganglion neurons. Neurosci. Res..

[57] Rimboeck J., Gruber M., Wittmann S. (2023). Is the In Vitro Observed NETosis the Favored Physiological Death of Neutrophils or Mainly Induced by an Isolation Bias?. Int. J. Mol. Sci..

[58] Pérez-Figueroa E., Álvarez-Carrasco P., Ortega E., Maldonado-Bernal C. (2021). Neutrophils: Many Ways to Die. Front. Immunol..

[59] Kim S.Y., Kim N.K., Baik S.H., Min B.S., Hur H., Lee J., Noh H., Lee J.H., Koo B.-N. (2016). Effects of Postoperative Pain Management on Immune Function After Laparoscopic Resection of Colorectal Cancer: A Randomized Study. Medicine.

[60] Sarenac O., Lazovic R., Vukcevic B., Lazovic J.R., Palibrk I.G. (2023). Impact of Perioperatively Administered Amino Acids, Lidocaine, and Magnesium on Inflammatory Response and Pain Associated with Abdominal Surgery: A Prospective Clinical Study. Med. Sci. Monit. Int. Med. J. Exp. Clin. Res..

[61] Yokoyama M., Nakatsuka H., Itano Y., Hirakawa M. (2000). Stellate ganglion block modifies the distribution of lymphocyte subsets and natural-killer cell activity. Anesthesiology.

